# A generating technique and knowledge representation of multiple-answer problems for learning with solving knowledge

**DOI:** 10.1007/s41039-015-0005-1

**Published:** 2015-06-23

**Authors:** Noriyuki Matsuda, Hisashi Ogawa, Tsukasa Hirashima, Hirokazu Taki

**Affiliations:** 1 Faculty of Systems Engineering, Wakayama University, 930 Sakaedani, Wakayama, 640-8510 Japan; 2 The Joint Graduate School in Science of School Education, Hyogo University of Teacher Education, 942-1 Shimokume, Kato, Hyogo 673-1494 Japan; 3 Department of Information Engineering, Hiroshima University, 1-4-1 Kagamiyama, Higashi-Hiroshima-shi, Hiroshima 739-8527 Japan

**Keywords:** Multiple-answer problems, Knowledge acquisition, Problem solving, Knowledge-based system

## Abstract

**Background:**

Erroneous answers in multiple-answer problems not only make the correct answer harder to determine but also indicate why the correct choice is suitable and the erroneous one a mistake when compared to the correct answer. However, it is insufficient to simply create erroneous answers for this purpose: explanations of these answers are also required. Preexisting studies examining functions for generating erroneous answers and their explanations based on this approach are abundant. Nevertheless, a major bottleneck has formed in this research body concerning the related specialized knowledge descriptions that are required for the generation function.

**Methods:**

This paper focuses on the notion that it is easy for teachers skilled in problem solving to express specific problems in written form and amend incomplete knowledge. Furthermore, it examines a method of constructing knowledge while generating and updating knowledge from specific problems.

**Result and Conclusion:**

The suitability of the proposed method was verified by examining actual knowledge constructed by the research subjects.

## Background

The stronger a learner’s naive understanding and determination, the more willing they are to independently accept defects (errors) in their knowledge and make corrections (learning from mistakes); hence, it is important to provide appropriate support (scaffolding) for this. Teaching materials play a significant role in guiding learners to “the right” realization and subsequently toward correcting mistakes. This is particularly true in cases of independent study, in which materials must guide learners in this “right” direction without interfering with the learner’s ability to realize, understand, and correct mistakes (Perkinson 1984). In recent years, teaching materials for independent study (such as those for e-learning) have been used in various fields. For instance, materials containing problems that prompt learners to confirm their understanding of fundamental facts and relations, and facilitate learning through the repetition of simple learning exercises, have emerged. Multiple-answer problems are typically the question form used when setting questions in these types of materials.

Multiple-answer problems entail presenting a learner with multiple answers and directing them to choose the correct one. The erroneous answers provided utilize either an approach whereby the erroneous answer cannot be identified correctly as a distractor (denoted a meaningless erroneous answer henceforth) or an approach in which the erroneous answer reflects a potential mistake made by the learner (denoted a meaningful erroneous answer henceforth). In the former case, which is common in the context of a language or aptitude test, the quality of the correct selection is more significant than the content imbedded within the erroneous answers; each selection apart from the correct one is equally erroneous, and an explanation of the correct choice is sufficient. Therefore, problems using these types of erroneous answers can be created with relative ease. However, in the latter case, the learner commits the error expressed in an erroneous answer by selecting it; consequently, it becomes possible to provide highly refined support according to the ascertained errors. When considering the effects of learning, it is preferable to create teaching materials with multiple-answer problems that utilize meaningful erroneous answers; this research proposes a technique for the automatic generation of such answers.

In multiple-answer problems, the provision of text explaining why a selected answer is erroneous plays an important role in leading independent learners to an accurate understanding and toward correcting their mistakes. Munby (1968) asserts that erroneous answers should prompt learners to make careful considerations before making their selection; in this scenario, learner instruction is affected by the selected choice, and texts explaining the erroneous answers are called explanations of erroneous answers. The authors believe that there are two prerequisites for erroneous answers and their explanations to guide learners in the right direction: (1) the choices should reflect a typically occurring learning error envisaged by the teacher, and (2) the learner must closely examine the differences between the erroneous and correct answers. The former is important as it ensures that learners make “good” mistakes and recognize them accordingly, while the latter prevents pupils from adopting misguided approaches when correcting mistakes. Erroneous answers meeting the aforementioned conditions are distinct from those selected at random based on the mere condition that they differ from the correct one.

When teaching materials use multiple-answer problems, such as for e-learning, a sufficient quantity of questions must be created. The work required to accomplish this is burdensome; however, an alternative solution involves technology that mechanically generates multiple-answer problems. A condition for answers appropriate for multiple-answer problems is that they are sufficiently confusing so that the learner cannot judge whether the erroneous answers are clearly different from the correct answer. Automatic generation mechanisms that use text processing and statistical analysis (cf. Moser et al. 2012; Correia et al. 2010; Gotoh et al. 2010), a corpus thesaurus of general knowledge within a particular field (cf. Sumita et al. 2005; Lin et al. 2007; Brown et al. 2005), and domain ontology (cf. Holohan et al. 2005; Mitkov et al. 2006; Alsubait et al. 2012; Papasalourosa et al. 2011) have been suggested. While these techniques can automatically generate large numbers of problems at a single time, the erroneous answers generated by them serve a different purpose than those proposed here. Conversely, there is an approach that attempts to automatically generate problems by employing the problem-solving abilities of machines (Funaoi et al. 2006). For example, GRAMY system (Matsuda and VanLehn 2004) can solve complex problems by precisely describing the problem-solving knowledge required for geometric-proof problems, from which it generates high-quality guidance according to the learner’s specific learning situation.

It is possible to implement these approaches by fully investigating methods of expressing knowledge in each respective field and then to create knowledge expressions that completely express the problems’ solutions. However, creating the descriptions of such knowledge is non-essential work in terms of the person solving the problems and can be considered work performed so that a machine can read and process the knowledge. In knowledge expressions, for example, there are collections of if-then rules that satisfy predicate expressions and OR relations. Even if a person is an expert in problem solving in a certain field, it is not necessarily true that they will be skilled at describing their knowledge for the machine. This point can be a major hurdle for teachers creating teaching materials for independent study. Richards and Compton (1998) discuss the relationship between ripple-down rules (RDR) and the expression of knowledge for problem solving. RDR recognize that experts in areas where it is difficult to describe machine-processable knowledge are highly proficient at explaining concrete problems and correcting errors related to problem-solving knowledge that has already been expressed. Therefore, RDR propose a mechanism whereby multiple experts can update existing knowledge.

Considering the above points, we propose a mechanism for generating multiple-answer problems with meaningful erroneous answers and an explanation of the errors for learning purposes. This undertaking requires our road map is below:

i. *Establishment of core technique*: to ascertain a technique of generating multiple-answer problem and its knowledge representation of the solution
ii. *Design of effective functions*: to define meaningful erroneous answer by analyzing the structure of actual problems
iii. *Establishment of generic technology*: to establish generic technology for generating multiple-answer problems for learning with meaningful erroneous answers

This paper occupies the first step (i) which is core technique. It is important to verify establishment of this core technique. Steps (ii) and (iii) are applications of the core technique to enhance learning. Our approach facilitates knowledge expression of problem solving and provides a system to assist in describing specific knowledge. This approach depends on both specific some domain and simple knowledge to describe. However, in constructing multiple-answer problems, we usually specify some concrete domain for the problems, an experiment to investigate the validity of simulation of erroneous solution (SES) generated problems and confirm expressed knowledge.

This research attempts to achieve “perturbation”, which deviates slightly from the correct problem solution, albeit within a cognitively appropriate range. Knowledge comprised of simple horn clauses described in programming languages like Prolog was used to automatically generate meaningful erroneous answers and explanations. DeJong and Mooney (1986) utilized Prolog for knowledge expression in their explanation-based learning (EBL) module, upon which the aforementioned SES is based. The SES combines cognitively appropriate perturbations with a problem-solving module that employs simple horn clauses to generate erroneous answers (Ogawa et al. 2013). A preliminary experiment to generate erroneous answers and their explanations using descriptions of knowledge (which is discussed in greater depth later) revealed that although SES is capable of generating explanations for meaningful erroneous answers, it cannot generate explanations of those answers and occasionally produces meaningless selections.

Next, we focus on the notion that it is comparatively easy for teachers attempting to describe problem-solving knowledge for multiple-answer problems to explain specific problems and update the expressed knowledge. In this context, preexisting knowledge does not exist in the form of expressions, and therefore, two steps must be taken. First, in function 1, the knowledge expressed in Prolog from the two or three specific questions created by the teachers is generated; this is then used in function 2 to update preexisting knowledge. Based on the knowledge being edited in function 3, update work is performed in tandem as SES is used to generate multiple-answer problems. Later, embodiment in abstract rule (EAR) is discussed, our proposed method of expression. Through an experiment, we confirm whether subjects can adequately describe SES knowledge.

## Methods

**Fig. 1:**
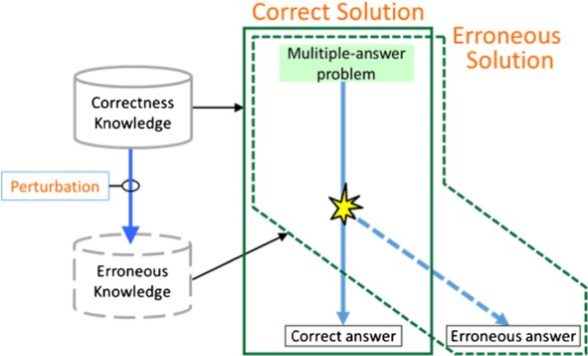
Outline of SES. SES provides erroneous answers and explanations for the errors with perturbation of correctness knowledge. Perturbation slightly alters correctness knowledge within a cognitively appropriate range

Figure 1 shows an outline of SES in which the knowledge to derive correct and incorrect answers is termed correctness and erroneous knowledge, respectively. SES generates the correct answer using the problem-solving device and then uses the rules that were utilized in deriving that answer to automatically generate the text explaining it. Next, perturbation is performed from the correctness to erroneous knowledge using the perturbation operator. Erroneous answers are generated from the erroneous knowledge, and explanations of the answers are generated using the rules that were utilized to derive them.

A condition of the multiple-answer problems discussed in this paper is that the answers comprise one correct answer and multiple erroneous ones that reflect mistakes learners typically make. Furthermore, because SES is unsuitable for creating small quantities of multiple-answer problems, it is intended for those who want to generate large quantities of multiple-answer problems that can be solved using procedural knowledge. Furthermore, as it generates problems using the space from Prolog horn clauses, the multiple-answer problems are limited to questions about simple facts and the relations between them.

### The problem-solving device

**Fig. 2:**
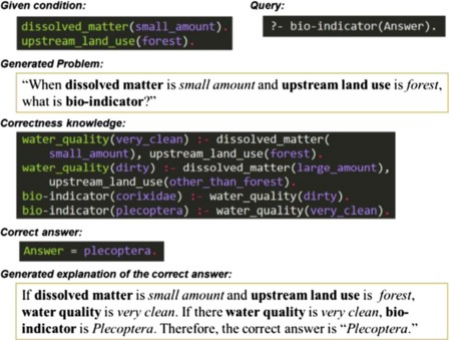
Correct solution using SES. SES generates a problem from the given conditions and a query. SES then generates a correct answer and explanation from the correctness knowledge based on EBL

**Fig. 3:**
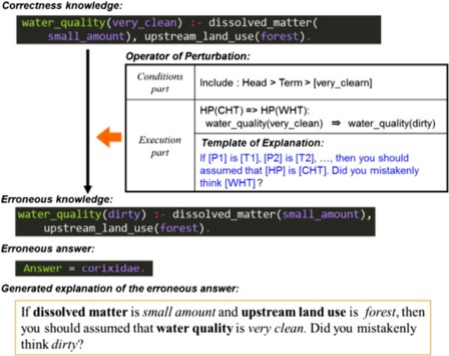
Erroneous knowledge by perturbation. A perturbation operator generates erroneous knowledge, and SES generates an erroneous answer and explanation corresponding to the correctness answer and explanation. The operator consists of a conditions component for the replacement of correctness knowledge and also an execution component. The execution component has a template of explanation. When clauses included in the solution are added into the template, an explanation is completed. This method is based on EBL

Figure 2 shows the process of generating a correct answer and its explanation using the problem-solving device. The problem conditions are facts given to the respondent through the problem text, while the question conditions are facts that become question items for the problem condition. Correctness knowledge entails the rules describing the relations between facts, and each is described using Prolog. Correctness (or erroneous) knowledge includes a list of simple horn-clause elements, and as shown in Fig. 3, the argument for the predicate is restricted to one item; the variables, list structure, and functions outside of the predicate logic’s scope are not included in the argument. Consequently, by applying descriptions in Prolog that use a simple structure, it becomes easy to generate the explanatory text; additionally, it becomes possible to easily select the question and problem condition from the correctness knowledge.

The problem-solving device first derives the correct answer from the problem condition, question condition, and correctness knowledge. Next, applying the problem and question conditions to the problem-text template generates the problem text. Finally, the text explaining the correct answer is generated by applying a list of clauses in the solution derivation process with the text template to explain the correct answer.

### The perturbation operator

SES uses erroneous knowledge to perform perturbation of correctness knowledge in the problem-solving device. The perturbation is a projection of the mistake types made by learners; in the expressions of perturbation, the perturbation operators expressing differences between the learner’s erroneous and correctness knowledge are used. The perturbation operator expresses the differences between the correct method of solving a problem and the learner’s erroneous method; in parallel, this difference is expressed in the explanatory text. As shown in Fig. 3, the perturbation operator is composed of a conditions and operations component. The conditions component is necessary for the perturbation operator to substitute rules, while the operations component is composed of the knowledge operator, which expresses the conversion of correctness knowledge to erroneous knowledge by the operator, and the explanatory-text template, which explains this operation.

The goal of this research is to automatically generate large quantities of meaningful erroneous answers and corresponding explanatory texts. To achieve this, a sufficient number of perturbation operators must be created. Hence, in addition to the user’s manual perturbation, whereby the described erroneous concepts are manually inputted, it is assumed that automatic perturbation, in which a machine automatically generates concepts from solution-method knowledge, will be effective for this task. Automatic perturbations do not depend on a specific field, and by describing the relations between the attribute values in advance, it becomes possible to automatically generate a perturbation by reversing the attribute value relation of perturbation from dissolved matter (a lot) to dissolved matter (a little). However, automatic perturbation cannot be utilized for mistakes specific to a certain field, and in these instances, manual perturbation is required. Therefore, by making use of both automatic and manual perturbations, it is possible to automatically generate large quantities of meaningful erroneous answers.

### Generation of explanations of erroneous answers

**Fig. 4:**
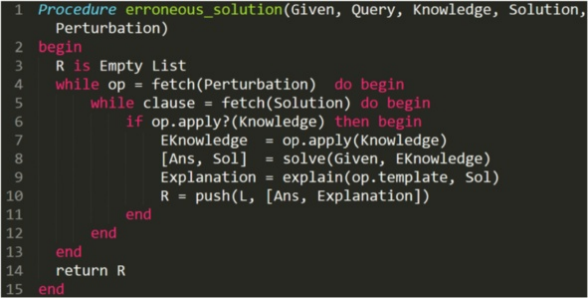
Correct solution using SES. SES generates a problem from the given conditions and a query. SES then generates a correct answer and explanation from the correctness knowledge based on EBL

By substituting the erroneous rules used for the derivations into the template for the explanation of erroneous answers, the generation of explanations of erroneous answers that adequately explain the differences between the correctness knowledge and the learner’s erroneous knowledge is achieved. However, as the multiple-answer problems are generated using the problem space from the horn clauses, they are limited only to questions about simple facts and the relations between them. Figure 4 shows the algorithm for creating erroneous answers and their explanations. The algorithm functions according to the steps outlined below:

- Each operator’s (op) perturbation is repeated.
- If the op’s condition is satisfied within the solution, the following three steps are executed:

1. Erroneous knowledge is obtained by applying correctness knowledge to the op.
2. The erroneous answer and solution are obtained with the erroneous knowledge.
3. The solution is added to the op’s explanation template to generate an explanation of the erroneous answer

Using this method, it was confirmed that 48 and 128 types of problem texts/erroneous answers and their explanations could be generated, respectively, from the correctness knowledge, in addition to two perturbation operators. It was further confirmed that the automatically generated answers included meaningful erroneous answers.

### Prototype system configuration

**Fig. 5:**
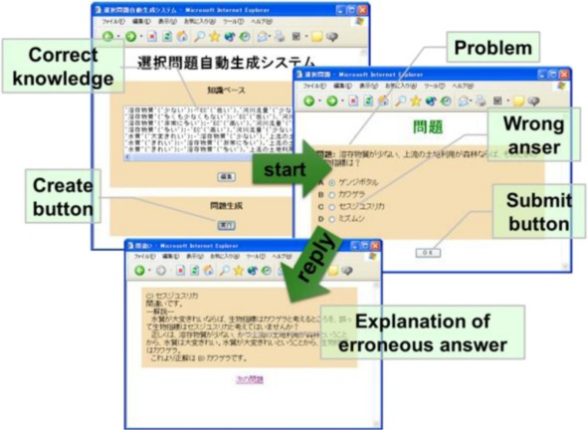
SES screenshots. The system first shows correctness knowledge and then generates a problem, after which the user (a learner) can make a selection and click the “submit” button. If the selection is wrong, an explanation of the error is provided

The prototype SES system was developed as web application using Perl and SWI-Prolog. The user inputs knowledge from the field of learning (correctness knowledge), along with a mistake typically made by the learner (perturbation operator). When a request to generate a problem is lodged with the system, it uses the perturbation operator to perturb the correctness knowledge with the erroneous knowledge after creating the correct answer and its explanation using the correctness knowledge; the erroneous knowledge is then used to generate erroneous answers and their explanations. Figure 5 contains screenshots from three windows: the first shows correctness knowledge, the second shows a problem, and the third shows the explanation of an erroneous answer.

### Evaluation experiment

A total of 2 surveys were administered to 2 water quality experts and 22 engineering course university students studying river water quality to determine the suitability of the proposed method for generating explanations of erroneous answers using SES. One of the experts is teaching at university.

#### Survey 1

*Method*: The two experts first inputted the correctness knowledge and perturbation operators into the system, after which the erroneous answers and their explanations were returned. Within a range where the results of the investigation would not be affected, they were given advice concerning the Prolog description method required when inputting correctness knowledge. From among the explanations returned, six were randomly selected and used. From the viewpoints of “Can the content of the erroneous answers’ explanations be understood?” and “Do the erroneous answers’ explanations sufficiently indicate the learners’ mistakes?”, the experts completed a questionnaire survey using a five-point Likert scale and free-entry responses. Additionally, they modified the erroneous answers’ explanations generated by the system to reflect a more appropriate explanatory text.

*Results*: The results for the first survey are shown in Tables 1 and 2. From the perspective of “Can the content of the erroneous answers’ explanations be understood?”, six answers were analyzed, and from among them, the experts selected, “I understand their meaning” and “Cannot say either way” for five and one of them, respectively. Additionally, from the perspective of “Do the erroneous answers’ explanations sufficiently point to the learners’ mistakes?”, they selected the choice, “They sufficiently point to them” for five of the six explanatory texts.

**Table 1:** Frequency of two experts’ answer against the question “Can the content of the erroneous answers’ explanations be understood?” in survey 1

5. I understand their meaning	4.	3. Cannot say either way	2.	1. I don’t understand their meaning
5	0	1	0	0

**Table 2:** Frequency of two experts’ answer against the question “Do the erroneous answers’ explanations sufficiently point to the learners’ mistakes?” in survey-1

5. They sufficiently point to them	4.	3. Cannot say either way	2.	1. They don't sufficiently point to them
5	0	1	0	0

*Considerations*: For the question, “Can you understand the content of the erroneous answers’ explanations?”, the expert who answered “Cannot say either way” justified his selection by noting that the explanatory texts were long and difficult to understand. This is likely because an identical erroneous answer was generated for three separate questions, and so, three solution methods for the error were reflected in the erroneous answer’s explanation. However, this paper’s objective is to automatically generate explanations of erroneous answers that explain the difference between the correct solution method and the learner’s incorrect solution method; consequently, this point is not considered problematic.

For the question, “Do the erroneous answers’ explanations sufficiently point to learners’ mistakes?”, the experts selected, “They adequately point to them” for five of the six explanatory texts. They provided affirmative answers for their reasoning such as, “The explanatory texts firmly grasped the combinations in which the learners were likely to make a mistake”. From this, it cannot be said that there were problems with the quality of the explanations generated using this method. Conversely, for one question, both experts selected the option, “Cannot say either way”.

Concerning the system-generated explanation of the erroneous answer, “If the water quality is clean, you should have assumed that the bio-indicators would be Plecoptera. Did you mistakenly think that the bio-indicator was Chironomidae?”, the experts stated that, “This only presents the knowledge that Chironomidae do not inhabit places with clean water” and “Even with a mistake in judgment, it is necessary to show the correct conditions and guide learners to this result”. Following the experts’ amendments, the sentence generated was “If the water quality is clean, you should have assumed that the bio-indicator would be the Plecoptera. Did you mistakenly think it was the Chironomidae that inhabits locations with very dirty water?”. Regarding the erroneous answer selected by the learner, they added an explanation that clarified the correct conditions to guide the learner to this erroneous answer. This is a problem with the explanation description template, and it can be resolved by adding descriptions to the template related to the error’s cause.

#### Survey 2

*Method*: The survey’s participants included 22 students; explanations of erroneous answers for three different problems were shown to each individual. The explanations for incorrect choices were generated using SES with knowledge inputted by the experts in survey 1. Next, individual, non-structured interviews were conducted in which the students were asked what they thought should be improved or amended to the erroneous answers’ explanations.

*Results*: The subjects identified the following eleven issues with the refined explanations:

1. It is uncertain whether the learner can follow the explanation completely in relation to the explanatory text’s main point.
2. Text explaining the correct solution method is necessary.
3. The language and sentences are difficult to understand.
4. It is necessary to consider factors such as the learner’s age and academic level.
5. It is necessary to provide conditions that guide learners to the correct result even in cases of mistaken judgment.
6. The explanatory texts indicate the incorrect points but do not explain the causes.
7. It is necessary to include elements that encourage learners to review the teaching materials.
8. Photographs and diagrams should be used in explanations.
9. The learner might have made a mistake other than the one indicated.
10. It is necessary to provide explanations that are more detailed in the absence of a knowledge base.
11. It is impossible to make appropriate judgments based on vague expressions such as “a little dirty” or “very clean”.

*Considerations*: Among the 11 points described above, 1–6 can be solved using the method described in this paper. Regarding points 1 and 2, it is possible to address these by integrating the correct answer and the erroneous answers’ explanations using the explanation of the correct answer generated before perturbation, as in the following example:

*Chironomidae is incorrect. If the water quality is clean, you should have assumed that the bio-indicator would be Corixidae. Did you mistakenly think the bio-indicator was Chironomidae? The correct answer is … (omission); if the water quality is dirty, the bio-indicator is Corixidae. Therefore, the correct answer is Corixidae.*

Concerning 3, the system can be improved by adding an editor function that allows one to modify the problem-text template. As for 4, allowing one to set the difficulty level for the problem and question conditions should facilitate the generation of erroneous answers corresponding to the learner’s proficiency. Points 5 and 6 were addressed in study 1 and are related to shortcomings in the explanatory-text template descriptions, which can be solved by changing the explanatory-text template describing the mistake’s cause.

At the current stage, it is difficult to address points 7–11; however, we do have some potential suggestions. For 7, the simple instruction, “Please review the problem text” could be added, although a function offering advice focusing on a specific part of the problem text would be difficult to include solely through this method, and further investigation is required. Addressing point 8 would necessitate including information in the form of figures and tables within the knowledge information; however, the knowledge editor would have to be redesigned for this purpose. While the issues highlighted in points 9–11 concern knowledge inputted by the experts, the cause of these problems can be attributed to limited expressible knowledge. By expanding the knowledge expressions handled by the system, it should be possible to address the aforementioned points.

### Summary of SES

SES was employed to generate erroneous answers by adding cognitively appropriate perturbations to a problem-solving device using a problem space from simple horn clauses described in Prolog. Based on the perturbations used to generate the erroneous answers, SES was proposed as a method for generating text explaining the answers. Results of surveys using the erroneous answers’ explanations confirmed that the automatically generated answers were meaningful. Moreover, the survey results suggested that the generated explanations were appropriate in that they could adequately point to and explain learners’ mistakes. Nevertheless, SES requires that knowledge be described using Prolog, and this is significantly burdensome for the user. Furthermore, there are concerns that the problems may not be correctly generated due to incorrect knowledge descriptions.

## Embodiment in abstract rule

To automatically generate multiple-answer problems in EAR, teachers update the problem-solving knowledge through a dialogue with the system regarding the knowledge, while also confirming the generated problems (see Fig. 6) (Ogawa, et al., 2011).

**Fig. 6:**
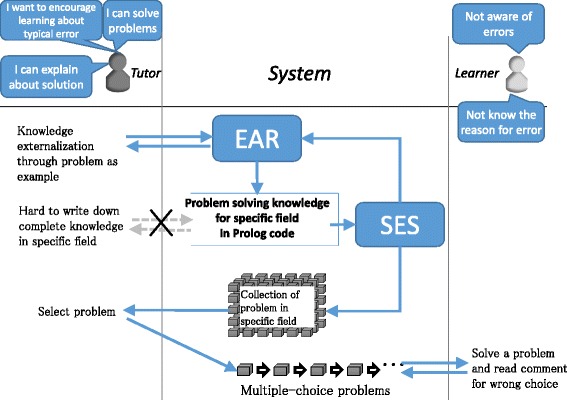
Overview of EAR knowledge externalization. A tutor can edit knowledge of the problem solution, after which he/she enters a concrete problem directly rather than by describing knowledge in Prolog. EAR uses SES to generate multiple-answer problems with current knowledge edited by the tutor. When the tutor completes editing in EAR, problems can be selected for learners

SES is used to automatically generate multiple-answer problems from descriptions of problem-solving knowledge written in Prolog. Even for teachers who can easily solve these problems, writing descriptions of the knowledge in Prolog is not an easy task. In EAR, knowledge is generated following the input of specific problems and then updated; using SES, knowledge is updated while the automatically generated problems are confirmed.

Figure 7 shows a sample problem generated with SES, while Fig. 8 shows an example of the knowledge expressed in Prolog. These figures contain scientific knowledge regarding water quality set by the teacher. Specifically, they demonstrate that the amount of dissolved matter is determined by upstream land use, flow volume, and electric conductivity (EC) and that the bio-indicators are determined by water quality. In this way, with knowledge as the rules, SES can obtain the correct answer by executing the Prolog program in which the facts are set as problem conditions.

**Fig. 7:**
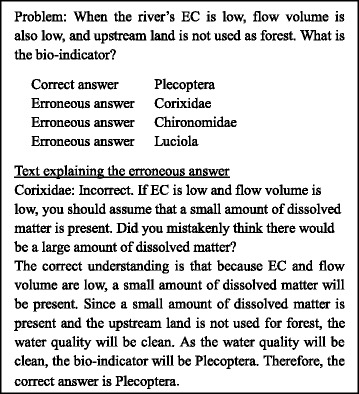
An SES-generated multiple-answer biology question

**Fig. 8:**
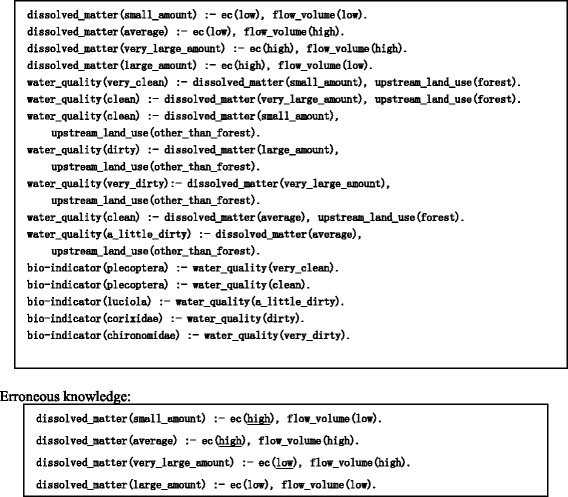
Correctness and erroneous knowledge for SES. “Correctness knowledge” regarding water quality set by the teacher is shown here. The following relations are demonstrated: that the amount of dissolved matter is determined by upstream land use, flow volume, and EC and also that the bio-indicators are determined by water quality. The teacher provides “erroneous knowledge” as well, which is thought to constitute particularly serious mistakes for learners. An opposite to correct relationship is demonstrated here in which EC is high and the amount of dissolved matter is small

Based on the process for deriving the correct answer, the explanatory text for the problem-solving process is generated by substituting the attribute name and value into the explanatory-text template. Additionally, the teacher submits the erroneous knowledge that is thought to constitute particularly serious mistakes for learners in Prolog. Figure 8 illustrates an opposite to correct relationship, namely, that when EC is high, the amount of dissolved matter is small. In SES, the rule that expresses perturbation is inserted prior to the correct one, the program is executed, and the erroneous answer and its explanation are obtained. This allows Prolog to prioritize and execute earlier rules. The following section describes the functions and respective procedures of EAR.

### Functions and procedures

EAR comprises the following three functions:

1. *Attributes generation*: expresses the attributes and attribute values of the knowledge possessed by the teacher and, after specific problems and a number of questions and answers are entered, outputs Prolog clauses.
2. *Relation editing*: the knowledge expressed in Prolog is graphed as it is created, and through editing operations in Prolog, rules are output.
3. *Problem confirmation*: using the Prolog being created, each generated problem is listed in SES and individual problem details are output.

Below, the specific operating procedures for each respective function with corresponding screenshots are provided. Concerning their order of use, after first performing the operations for attributes generation, users can freely use the functions in any sequence.

#### Procedure for the attributes-generation function

The following two procedures are required to create attributes and attribute values corresponding to the Prolog clauses of the inputted problem.

1. *Creating the base problem*. The teacher inputs the problem text, answers, and explanatory text as a specific example. This is called the base problem. The answers inputted become the attribute values. Since problems and explanatory texts are natural-language texts, they are not processed automatically. In a subsequent procedure, when the attributes and their values are created, processing is used to extract the necessary words and phrases from these texts. In Fig. 9, “EC”, “flow volume”, “small amount”, “dissolved matter”, “upstream land use”, “other than forests”, “water quality”, “dirty”, and “bio-indicators” are extracted.

**Fig. 9:**
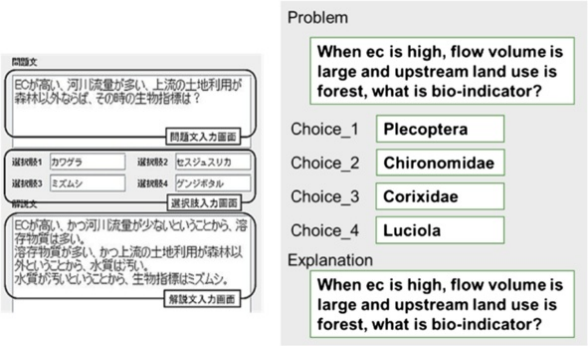
Base problem editor. The illustration on the *left* is a screenshot from the original Japanese interface; an English translation is provided on the *right*. This screen allows the tutor to enter a concrete problem
2. *Creating problems from the differences*. As the user continues to submit problems, they input only the difference with the base problem. At this time, EAR asks a question regarding the common attributes of the difference and requests input; it then generates attributes based on this input. In Fig. 10, the base problem attribute value of Plecoptera and the difference of Corixidae are derived from the bio-indicators.

**Fig. 10:**
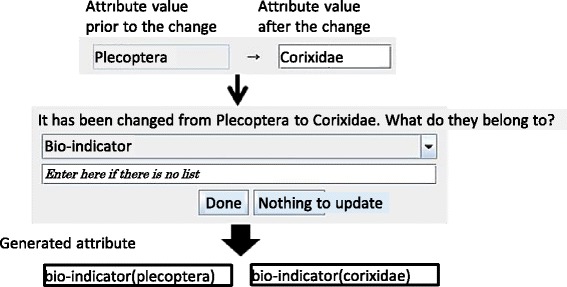
Attributes and values editor. To enter another problem, the tutor inputs the differences from the base problem shown in Fig. 7. This composite image was created by superimposing English words onto the original Japanese screenshot

SES designates the attributes presented and questioned in the problem text as “problem conditions” and “question conditions”, respectively. At this stage, one can set the question or problem conditions (or both the question and problem conditions) for the created attributes. Any attributes set as question and problem conditions to fulfill the question condition are changed by SES at random, and the remaining attributes are used as problem conditions to generate the problem. A problem is generated for each combination; non-specified attributes are used only in the problem-solving process and do not appear in either the problem text or answers. Next, the relations between the attributes and attribute values (the Prolog rules) are created. The following section describes the procedures encompassing this process in greater detail.

#### Procedure for the relation-editing function

1. *Creating the correct relations.* The relation between water quality and bio-indicators in Fig. 11 corresponds with the fundamental rules for knowledge in Fig. 8. These relations, including the knowledge for creating attribute relations, are expressed in one graph; the arrow from the problem to question condition indicates the relation between the attributes. For example, the knowledge in Fig. 8 is shown in Fig. 12, wherein the bio-indicators are set as question conditions, while flow volume, EC, and upstream land use are set as problem conditions. There are no settings for dissolved matter or water quality.

**Fig. 11:**
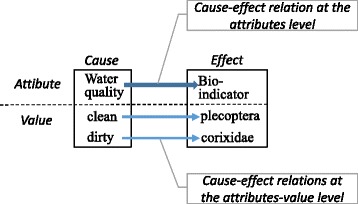
Attribute and attribute value relations. The tutor must identify relations at both the attribute and attribute value levels

**Fig. 12:**
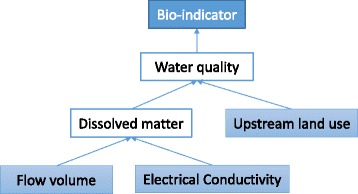
Graphical representation of attribute relations. The graph was created by a water quality specialist specifically for a junior high school lesson. During the lesson, students visited a nearby river and surveyed its bio-indicators and EC to determine the water quality. Here, “bio-indicator” is question condition while “upstream land use,” “flow volume,” and “EC” are problem attributes. “Water quality/dissolved matter” is an additional attribute related to the problem’s solution. Learners must determine these attributes independently

While referring to the knowledge in the graph, the teacher corrects incomplete knowledge. Specifically, they can perform the following editing tasks:

- Add, correct, or delete attributes
- Create new relations with other attributes
- Delete unnecessary relations
- Refer to, add, correct, or delete attribute values
- Change the display order of attribute values among attributes
- Create, correct, or delete relations between attribute values among attributes
- Refer to, add, correct, or delete perturbations

Consider the following example (see Fig. 13). When neither water quality nor dissolved matter is connected to any attributes, a relation between water quality and upstream land use is created. When this is determined, a new relation is created between both attributes, and it appears as a link in the graphical expression. In parallel, relations are created in a defined order between each of the respective attribute values. Teachers can edit the relation between attribute values when they want to change these new relations. In the initial editing stage, deficiencies must be supplemented, since neither a sufficient number of attributes nor relations have been created. In Fig. 14, the attribute value relation between bio-indicators and water quality is created and added.

**Fig. 13:**
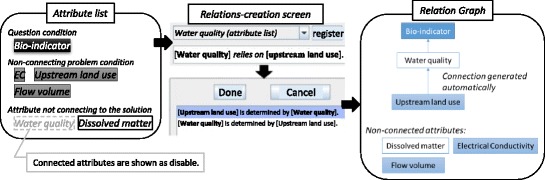
Editing of attribute relations. Figure 11 is the goal of this example. This composite image was created by superimposing English words onto the original Japanese screenshot

**Fig. 14:**
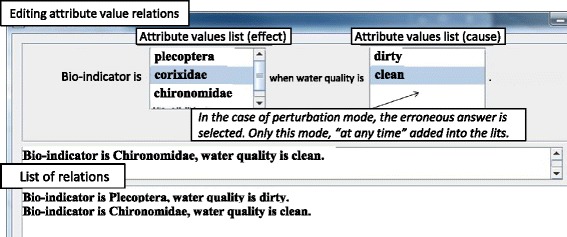
Editing attribute value relations. The tutor can create new relations by combining previously entered attribute values. Whenever the user needs to add an additional attribute or attribute value to create a relation, he/she can return to an earlier step. This composite image was created by superimposing English words onto the original Japanese screenshot

(2) *Creating erroneous relations*. In addition to correct relations, erroneous relations (i.e., perturbations) are also created. The operations to accomplish this are identical to those for the attributes and attribute values described above. To distinguish the perturbations screen from where correct knowledge is created, the perturbation mode is specifically labeled as such. For example, when an erroneous relation between Chironomidae and water quality is created, as in Fig. 14, the user can edit it as an attribute value relation. In the perturbation mode, however, multiple perturbations can be created simultaneously by selecting “at any time” as an attribute value’s wildcard.

In the process described above, the operations are not limited to making teachers continuously create correct relations and attributes, or relations and attributes that are significant for SES. For instance, looping relations and completely isolated attributes can be created with no relation to any attribute. When this is done, SES ignores relations and attributes other than the shortest relation from the problem to question condition without affecting problem generation.

#### Procedure for the problem-confirmation function

This function is used to confirm whether the knowledge being edited will ultimately be for the problem that the user intended. The user inspects the problems generated by SES and then returns to the attributes-generation or relation-editing function to perform editing as required. The information inspected by the user (the problem text, answers, and erroneous answers’ explanations) is identical to that shown in Fig. 6. For instance, the user might inspect how an added attribute relation affects the erroneous answers in a generated problem and the effects that the presence or absence of attributes has within a problem’s range. This inspection should result in editing activities that bring the knowledge closer to what the user intended.

### Evaluating the generated knowledge

In this section, the evaluation experiment is described. This experiment has already reported in the literature (Ogawa et al. 2013).

The following method was used to evaluate the extent that knowledge generated though an installation of EAR in Java could be deemed suitable. Materials explaining knowledge related to two fields (science [see Fig. 8] and junior high English) were distributed to test subjects in advance on a sheet of A4 paper (consists of 512 letters in Japanese and 1 figure). Participants were afforded an unlimited amount of time to read the materials. The English material entailed a basic problem asking for an appropriate verb based on the subject, person, and tense. A sample question is provided in the “Appendix” section. The test group used the EAR method (including the Prolog processing system and SES) to create knowledge, while the control group used a text editor, the Prolog processing system, and SES to create Prolog code. The obtained rules (relations) written in Prolog were later evaluated. The fundamental Prolog grammar was explained only to the control group.

#### Selection of test subjects

Test subjects were students in the faculty of engineering who had learned several programming languages. The set fields were first explained to the students during selection, and after the fields were understood, participants were tested to determine if they could judge problems appropriately; individuals were selected as test subjects only when they could categorize each problem correctly. During the test, participants were presented with three problems and asked to classify them into one of the following four categories:

1. A correct problem in which all knowledge is used neither excessively nor insufficiently.
2. An incorrect answer.
3. A problem in which knowledge not provided in the materials is used.
4. A problem in which knowledge provided in the materials is not used.

For example, the fourth category was the appropriate response for a problem in which water quality was not mentioned in the erroneous answers’ explanation. Seven students were chosen as test subjects following the selection process.

#### Evaluation items

The subjects’ knowledge descriptions were evaluated based on the precise and recall ratios of the attributes and attribute values. Regarding what these ratios express, a higher precise ratio indicates a lower number of erroneous relations, while a higher recall ratio indicates fewer omissions in the prepared materials. Two English and biology students comprised the test group, while a biology student and two English students comprised the control group.

#### Test method

The test comprised the following four steps:

1. Presenting the material to test-subject candidates and ensuring their understanding of it.
2. Selecting test subjects using the method described above.
3. Dividing test subjects into test and control groups and creating respective knowledge. No time limitations were enforced.
4. Switching the test/control groups established in step 3 and their respective fields before re-administering the test.

#### Results

Tables 3 and 4 show the precise and recall ratios for the cause/effect relations and generated problems, respectively. There are several ways to express knowledge, so the score denominator differed between subjects. The precise and recall ratios were calculated for the test and control groups, respectively, and a *t* test examining the differences in their mean values was performed. The results revealed a significant difference of 0.1 % in the precise (*df* = 6, *t* = 6.16) and recall ratios (*df* = 6, *t* = 3.90) for the attributes. As for the attribute values, a significant difference of 0.1 % was present in the precise (*df* = 6, *t* = 7.07) and recall ratios (*df* = 6, *t* = 18.0), with the test group being higher.

**Table 3:** Test results for attribute relations

Subject	Learning material	Experimental group		Learning material	Control group	
		Precise	Recall		Precise	Recall
A	English	3/3	3/3	Biology	0/7	0/4
		100 %	100 %		0 %	0 %
B	English	2/2	2/3	Biology	1/4	1/4
		100 %	100 %		25 %	25 %
C	English	3/3	3/3	Biology	2/5	2/4
		100 %	100 %		40 %	50 %
D	English	3/3	3/3	Biology	1/4	1/4
		100 %	100 %		25 %	25 %
E	Biology	4/4	4/4	English	2/3	2/3
		100 %	100 %		67 %	67 %
F	Biology	4/4	4/4	English	2/3	2/3
		100 %	100 %		67 %	67 %
G	Biology	4/4	4/4	English	3/5	3/3
		100 %	100 %		60 %	100 %
Mean		100 %	95.3 %		40.6 %	47.7 %
SD		0	12.5		25.5	33.6

**Table 4:** Test results for attribute values

Subject	Learning material	Experimental group		Learning material	Control group	
		Precise	Recall		Precise	Recall
A	English	32/32	32/32	Biology	0/7	0/10
		100 %	100 %		0 %	0 %
B	English	25/25	25/32	Biology	0/4	0/10
		100 %	78 %		0 %	0 %
C	English	27/27	27/32	Biology	2/6	2/10
		100 %	84 %		33 %	20 %
D	English	29/29	29/32	Biology	1/6	1/10
		100 %	91 %		17 %	10 %
E	Biology	10/10	10/10	English	2/3	2/32
		100 %	100 %		67 %	6 %
F	Biology	12/12	12/12	English	1/6	1/32
		100 %	100 %		17 %	3 %
G	Biology	10/10	10/10	English	6/10	6/32
		100 %	100 %		60 %	19 %
Mean		100 %	93.3 %		27.7 %	10.3 %
SD		0	9.17		27.0	9.39

#### Considerations

The discrepancy between groups could be attributable to a difficulty in describing knowledge regarding problem-solving areas in Prolog despite possessing sufficient knowledge of said areas. Although the control group could describe a number of relations, complete problem-solving knowledge is required to operate SES and generate problems, in cases where even a single relation is missing operation cannot occur. The researchers believe that EAR can potentially address this concern. Despite the fact that both test subject B and C’s precise and recall ratios were 100 % for relations at the attribute level, their recall ratios declined for relations at the attribute value level. Regarding the cause of this, the second-person singular “you” concurrently expressed the second-person plural; although this knowledge requires two items, participants only seemed capable of constructing descriptions for one. Upon examining the control group’s descriptions, there were no mistakes in Prolog grammar; however, missing attributes and contradictions were often generated in the relations. Hence, developing an awareness of these defects within the mechanism provided in the test-group environment was easy. Nevertheless, it was impossible to specify which function or sequence of operations was responsible for the effects.

## Results and discussion

A method of expressing problem-solving knowledge required for a machine to automatically generate multiple-answer problems was presented. The generated problems were intended to focus learners’ attention on recognizing their mistakes and the differences between the correct answers and their own incorrect answers. Even if a teacher is skilled in problem solving in a relevant area, it can be difficult for them to write descriptions of their knowledge as a collection of predicates and rules in Prolog. Assuming this is the case, it is still comparatively easy for instructors to write specific problems and correct deficiencies in the knowledge that has been expressed, and this is the focus of the design proposed in this paper. An evaluation of the knowledge generated upon installing and using EAR was conducted, which provided suggestions concerning the suitability of the proposed method.

## Conclusions

In establishing a technology for generating multiple-answer problems for learning with meaningful erroneous answers and explanations, this paper suggests employing core technique SES to generate multiple-answer problems according to their solutions using Prolog. EAR assists in describing knowledge of the problem solutions. Our approach requires descriptive problem-solving knowledge and a specific domain to be supplied in a Prolog Horn clause. The problems automatically generated by SES were confirmed to include meaningful erroneous answer and knowledge descriptions. Future research should focus on defining meaningful/meaningless erroneous answers by thoroughly analyzing the structure of actual utilized problems. This will help to elucidate the technique’s limitations and lead to the identification of additional problems and solutions. Finally, we need to establish generic technology for generating multiple-answer problems for learning with meaningful erroneous answers based on the core technique.

## Appendix

The following is a sample problem provided to study participants. It is an example of a problem wherein knowledge not shown in the materials is used.

Problem: When a sentence subject is “I” select the verb.

Correct answer: “Am playing”.

Erroneous answer: “Play”, “Is playing”, “Will play”.
